# Incidence of Hepatocellular Carcinoma in Southeast Iran

**Published:** 2010-12-01

**Authors:** Sodaif Darvish Moghaddam, Ali Akbar Haghdoost, Seyed Hamed Hoseini, Rashid Ramazani, Mohammad Rezazadehkermani

**Affiliations:** 1 Department of Internal Medicine, Afzalipour School of Medicine, Kerman University of Medical Sciences, Kerman, IR Iran; 2 Physiology Research Center, Department of Epidemiology and Biostatistics, Kerman University of Medical Sciences, Kerman, IR Iran; 3 Department of Epidemiology, School of Health, Shiraz University of Medical Sciences, Shiraz, IR Iran; 4 Deputy for Non Communicable Diseases of CDC, Iran Ministry of Health and Medical Education, Tehran, IR Iran; 5 Afzal Research Institute (NGO), Kerman, IR Iran

**Keywords:** Incidence, Hepatocellular carcinoma, Kerman, Iran

## Abstract

**Background and Aims:**

Hepatocellular carcinoma (HCC) is a well-known consequence of chronic liver disease (CLD). The aim of this study was to extract the HCC incidence rate in the province of Kerman, located in southern part of Iran, and compare the data with other parts of the country.

**Materials and Methods:**

All medical records related to HCC were collected through hospitals or outpatient services in public or private centers. The records of all oncology, radiotherapy, and pathology centers in Kerman province were actively searched between 1999 and 2006. The annual incidence of HCC around the country was calculated, using the national cancer registry database provided by the Health Ministry of IR Iran from 2005 to 2006. Using Stata version 8, the crude and age-sex-standardized annual incidence rates were computed.

**Results:**

The crude annual incidence rates of HCC per 100,000 persons in Kerman and Iran were 0.522 (95% CI = 0.238- 0.88) and 0.199 (95% CI = 0.167-0.234), respectively. When adjusting for age and sex, the annual incidence rates of HCC in Kerman and Iran were 0.7 (95% CI = 0.4-1.1) and 0.2 (95% CI = 0.2-0.3) per 100,000 persons, respectively (P<0.01).The mean age of patients in Kerman was around 5.5 years younger than other parts of Iran (56.17 ± 18.32 years versus 61.68 ± 14.62 years; P=0.004).

**Conclusions:**

In general, the incidence of HCC is not very high in Iran; however, the higher incidence of HCC in Kerman and also the lower age of onset mandates further research to detect HCC’s risk factors in this part of country.

## Introduction

Hepatocellular carcinoma (HCC), a malignant tumor originating from hepatocytes, is the sixth-most prevalent cancer worldwide. Furthermore, due to its poor prognosis, it is also the fourth leading cause of death related to cancer [1]. Although HCC is not generally common, it is an intractable disease [2]. Therefore, we have to pay special attention to the risk factors of HCC in order to decrease its burden on society. There is a great variation in the incidence of HCC between and even within countries. These differences might be due to regional variations in exposure to HCC risk factors. The annual incidence of HCC has been estimated to be high in Africa (24/100,000) and eastern areas of Asia (35/100,000); whereas its incidence is low in North America, Western Europe, and the Middle East (3-4/100,000) [3]. Although Iran is located in a low-risk region with an annual incidence well under 5 per 100,000 [2], we could not find specific information about its distribution within the country. Because the main known HCC risk factors vary around the country, it is reasonable to presume that its incidence also varies significantly. Some of the potent risk factors of HCC are now well-documented and include chronic viral hepatitis, cirrhosis, alcohol abuse, and Aflatoxin exposure [4]. However, other risk factors, such as smoking, obesity, and diabetes, are also associated with increased risk of HCC [5][6]. We did not find any specific information about the epidemiology of HCC in the literature on the Iranian population. There are a few published papers about the epidemiology of HCC risk factors such as Hepatitis B [7][8] and Hepatitis C [9] and their geographic distributions, but the incidence and geographical pattern of HCC are not clearly defined. The Iranian government’s Department for Fighting Cancer includes a cancer registry program, which published its first report in 1986. In 1999 the executive guidelines for cancer registry were revised and the second report was published; however, it is thought that the second report  captured only 18% of new cases. The last report, which is based on data from 2005, is estimated to reflect around 80% of new cancer cases in the country [10]. Kerman is a very wide province in southeast Iran, with a population of approximately 2.6 million. Traditionally, the lifestyle of its inhabitants, such as their diet, might increase the risk of HCC. For instance, this province produces more than 60% of the country’s pistachios, which used to be a main source of dietary aflatoxin exposure. In addition, viral hepatitis used to be more or less common in this province, but generally not greater than the national average [8][11]. There are also two reports about the prevalence of positive HBs antigen in pregnant women in the Kerman province, and their estimated prevalence seems to be the same as the national figure [12][13]. Based on the above explanation, we explored the data from the national cancer registry and also the province’s population-based registry in order to generate an acceptable annual incidence.risk of HCC in Kerman and compare this figure with the national incidence.

## Materials and Methods

We searched all databanks of cancer cases extensively, including all pathology, oncology, and radiology centers and medical documents and death registries within the Kerman province between 1999 and 2006. In this search, we extracted the data of all patients with any type of liver cancers to maximize our sensitivity to the detection of HCC. In addition, we searched the national cancer registry databank to find any possible Kermanian cases recorded in the data of other provinces. We excluded duplicate records by using the full names of cases. In order to compare the incidence of HCC in Kerman with the incidence in other areas in the country, the national and provincial figures in a few selective provinces were extracted from the cancer registry database. We selected six provinces because they have the most complete data in their cancer registry databanks (Esfahan, Razavi Khorasan, Khoozestan, Golestan, Ardebil, West Azerbaijan, Tehran, Fars, and Guilan). Because we expect the cancer registry in these provinces  to have maximum sensitivity, the estimated incidence in Kerman (data from different sources) is more comparable with the incidence in these selected provinces. The data were computerized using CanReg Software (produced by Iran’s Ministry of Health through its cancer registry program). The statistical analysis was performed using Stata (version 8). The total populations of provinces and their age and sex distributions were extracted from the data from the Iranian national census in 2006. The Standardized incidence of HCC was calculated at both the provincial and national levels using WHO’s world standard population. The 95% confidence intervals of the incidences were computed using a Poisson distribution and were compared using a Poisson regression model.

## Results

**Figure 1:**
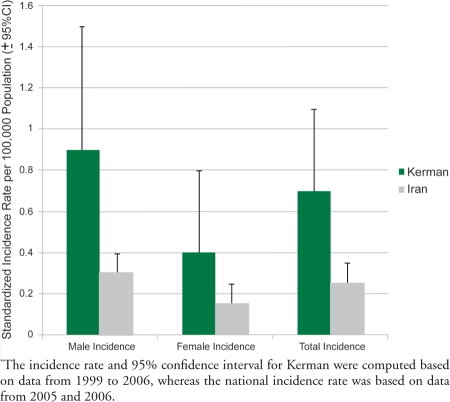
The Annual Icidence of Hepatocellular Carcinoma in Kerman and Iran Classified By Sex

**Figure 2:**
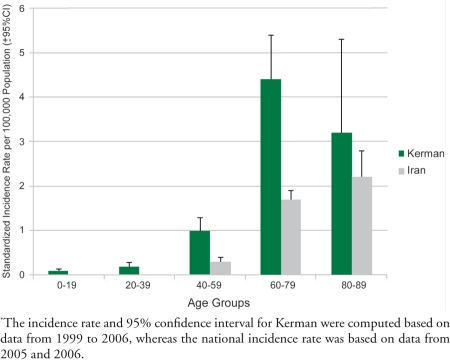
Hepatocellular Carcinoma Incidence in Different Age Groups

We found the records of 333 cases labeled as liver tumor in Kerman between 1999 and 2006; among them, only 95 cases had confirmed HCC diagnosis; others were mostly metastases of other cancers to the liver. Only two new HCC cases were added to our records from the national cancer registry databank; these two cases sought their treatments outside of the Kerman province. The crude annual incidence risk in Kerman was 0.522 (95% CI = 0.283-0.88) per 100,000. After adjusting for age and sex, the standardized risk was 0.7 (95% CI = 0.4-1.1) per 100,000. The standardized risk in males was 0.9 (0.3-1.5) and in females was 0.4 (0.0-0.8). During 2005-2006, 484 cases of liver tumors were recorded in the cancer registry database around the country. Among them, 279 records had a confirmed diagnosis of HCC. The crude annual incidence risk of HCC in Iran was 0.199 (95% CI = 0.167-0.234) per 100,000. After adjusting for age and sex, the standardized risk was 0.2 (95% CI = 0.2-0.3) per 100,000. The standardized risk in males w   0.3 (0.3-0.4) and in females was 0.2 (0.1-0.2). According to the data above, the relative risk of HCC incidence in the male population in Kerman and Iran was 2.35 (95% CI = 1.52-3.62) and 2.08 (95% CI = 1.46-2.96), respectively. The annual incidence rates of HCC in provinces with the maximum sensitivity in their cancer registries were much lower than the incidence rate in Kerman. Specifically, the incidence rate ranged from 0.3 (Tehran) to 0.08 (in Ardabil and Guilan), and it was slightly more than 0.5 in Kerman (Table 1). We found a significant difference between the annual risk in Kerman with the annual risk in Iran as a whole (P<0.01). This difference was observed in both males and females (Figure 1). In addition, upon examining the age distribution of cases in Kerman and in Iran (Figure 2), we found that the Kermanian cases were significantly younger. Specifically, in Iran the mean age was 61.68 years (SD= 14.62), whereas in Kerman the mean age 56.17 years (SD=18.32; P=0.004).

**Table 1:** HCC Cases Reported in Selected Provinces from 2005 to 2006

**Province**	**Mean number of annual HCC Cases^a^**	**Crude Annual Incidence per 100,000 (Poisson 95% CI)**
Kerman(Population Based Case Finding)	13.8	0.522 (0.283-0.88)
West Azarbaijan	8	0.222 (0.095-0.437)
Isfahan	9	0.197 (0.090-0.375)
Tehran	41.5	0.313 (0.226-0.423)
Razavi Khorasan	11.5	0.215 (0.111-0.375)
Khoozestan	7.5	0.187 (0.080-0.369)
Guilan	2	0.083 (0.010-0.30)
Fars	12.5	0.300 (0.160-0.513)
Golestan	3.5	0.247 (0.0674-0.633)
Ardebil	0.5	0.0814 (0.0020-0.454)

^a^ The incidence rate and 95% confidence interval in Kerman were computed based on data from 1999 and 2006, whereas the national incidence rate was based on data from 2005 and 2006.

## Discussion

This study found that the risk of HCC was higher in the Kerman province compared to the risk in Iran as a whole in recent years. This greater risk was observed for both males and females. In addition, the Kermanian cases tend to be younger than the Iranian cases. According to international classifications, areas such as the United States, Canada, and Scandinavia (which have incidence rates under 5 per 100,000) are considered low-risk areas [2]. According to the findings in this study, Iran should also be classified as a low-risk area in the world. There are a few possible factors that are thought to be associated with the low incidence rate of HCC in Iran, including a low incidence of alcoholic cirrhosis due to the legal and religious restrictions of alcohol consumption. Hajiani and colleagues found that less than 3% of HCC cases in the south of Iran were associated with alcohol consumption; this report also argued that Hepatitis B was the most common risk factor for HCC in their sample [14].

Moreover, relatively low prevalence rates of hepatitis B and C in Iran could be another explanation for the low rate of HCC. According to a WHO report, an estimated 3% of the world population have HCV [9]. The available data about the epidemiology of hepatitis C in Iran reveal a prevalence of less than 1% in the general population [9]. The prevalence of chronic hepatitis B in the general Iranian population is around 3% [7]. Reports from a transfusion organization showed that the prevalence of Hbs antigen among blood donors was approximately 6 per 10,000 samples, whereas this number in the Kerman province was around 9 per 10,000. However, since 1990, the hepatitis B vaccination has been a routine part of a national vaccination program for infants; therefore, we expect further declines in the prevalence of hepatitis B in the coming decades [15]. Although the incidence of HCC seems to be low in Iran in general and in the Kerman province as well, it seems that the incidence of HCC is much higher in Kerman compared to other parts of Iran. It should be mentioned that the incidence of other main upper GI cancers, gastric and esophageal cancers, were relatively low in the Kerman province [16], which is very different from the pattern of HCC. As mentioned before, the data used in this study suggest that the prevalence of viral hepatitis in Kerman is consistent with the national figure. The possible cause of increased risk of HCC in Kerman could be due to greater exposure to dietary aflatoxin in the past. Cheraghali et al. investigated the presence of aflatoxin B1 in 10,068 samples of Iranian pistachio, and they detected aflatoxin B1 in 3,699 samples (36.7%) [17]. Indeed, studies have pointed to the synergistic effect of aflatoxin and hepatitis in the development of HCC [18]. However, due to some issues, such as improvements in people’s lifestyles, turning attention away from the preservation of high-quality pistachio, and clearer definitions “health programs” by the Health Ministry of Iran, people’s exposure to aflatoxin have decreased conderably in recent decades. Some portion of the lower HCC incidence rate in Iran as a whole than in Kerman could be due to the low accuracy of the national cancer registry compared to the sensitivity of our multiple sources of active case findings in Kerman. However, because the diagnosis of HCC is not difficult, particularly in the advanced stages, we do not expect that this explanation by itself can explain such a wide difference between the incidence rates in Iran and Kerman. We also compared our estimates with those in provinces with high-quality cancer registries (Table 1), which showed that the crude annual incidence rate of HCC in Kerman was the highest of all provinces. In addition, in order to minimize this error, we only used the data from the last 2 years to estimate the national incidence rate. In general, we expect that the quality of the national cancer registry has been improved in recent years. In conclusion, we believe that the incidence of HCC in Kerman is greater than the rate in the whole cuntry, although the rate is still very low compared to the global figure.
